# Neuroanatomical Alterations in Patients With Tinnitus Before and After Sound Therapy: A Combined VBM and SCN Study

**DOI:** 10.3389/fnhum.2020.607452

**Published:** 2021-01-18

**Authors:** Xuan Wei, Han Lv, Qian Chen, Zhaodi Wang, Chunli Liu, Pengfei Zhao, Shusheng Gong, Zhenghan Yang, Zhenchang Wang

**Affiliations:** ^1^Department of Radiology, Beijing Friendship Hospital, Capital Medical University, Beijing, China; ^2^Department of Otolaryngology-Head and Neck Surgery, Beijing Friendship Hospital, Capital Medical University, Beijing, China

**Keywords:** tinnitus, sound therapy, VBM, structural covariance network, fMRI

## Abstract

Many neuroanatomical alterations have been detected in patients with tinnitus in previous studies. However, little is known about the morphological and structural covariance network (SCN) changes before and after long-term sound therapy. This study aimed to explore alterations in brain anatomical and SCN changes in patients with idiopathic tinnitus using voxel-based morphometry (VBM) analysis 24 weeks before and after sound therapy. Thirty-three tinnitus patients underwent magnetic resonance imaging scans at baseline and after 24 weeks of sound therapy. Twenty-six age- and sex-matched healthy control (HC) individuals also underwent two scans over a 24-week interval; 3.0T MRI and high-resolution 3D structural images were acquired with a 3D-BRAVO pulse sequence. Structural image data preprocessing was performed using the VBM8 toolbox. The Tinnitus Handicap Inventory (THI) score was assessed for the severity of tinnitus before and after treatment. Two-way mixed model analysis of variance (ANOVA) and *post hoc* analyses were performed to determine differences between the two groups (patients and HCs) and between the two scans (at baseline and on the 24th week). Student-Newman-Keuls (SNK) tests were used in the *post hoc* analysis. Interaction effects between the two groups and the two scans demonstrated significantly different gray matter (GM) volume in the right parahippocampus gyrus, right caudate, left superior temporal gyrus, left cuneus gyrus, and right calcarine gyrus; we found significantly decreased GM volume in the above five brain regions among the tinnitus patients before sound therapy (baseline) compared to that in the HC group. The 24-week sound therapy group demonstrated significantly greater brain volume compared with the baseline group among these brain regions. We did not find significant differences in brain regions between the 24-week sound therapy and HC groups. The SCN results showed that the left superior temporal gyrus and left rolandic operculum were significantly different in nodal efficiency, nodal degree centrality, and nodal betweenness centrality after FDR correction. This study characterized the effect of sound therapy on brain GM volume, especially in the left superior temporal lobe. Notably, sound therapy had a normalizing effect on tinnitus patients.

## Introduction

Tinnitus is a very common otological disorder. Approximately 10% to 25% of the population is severely affected (Baguley et al., 2013; Bauer, 2018). Tinnitus reduces the quality of life for millions worldwide (Shore et al., 2016). Chronic tinnitus can cause a series of problems, such as sleep disturbances (Schecklmann et al., 2015), cognitive problems, depression (Dobie, 2003), and work disorders (Heller, 2003). Previous studies have shown that tinnitus can cause significant changes in brain function and structure, which are closely related to the clinical manifestations of patients (Ryu et al., 2016; Han et al., 2019a). Also, studies have shown that the brain structure and function of tinnitus patients have undergone significant remodeling (Schmidt et al., 2017; Chen et al., 2020). Therefore, it is very important to fully understand the abnormal brain nerve activity related to tinnitus.

In our previous research, tinnitus has been confirmed to be a symptom of abnormal resting-state fMRI (rs-fMRI; Han et al., 2015; Lv et al., 2016a,b, 2018). The brain regions involved include sound detection regions, such as the insula and hippocampus (van der Loo et al., 2011; Hofmeier et al., 2018), and auditory and nonauditory brain regions (Vanneste and De Ridder, 2012), such as the parahippocampal gyrus (Vanneste et al., 2011), posterior cingulate cortex (Vanneste et al., 2010), and anterior cingulate cortex (De Ridder et al., 2011). In recent years, the study of brain microstructure has also received increasing attention. Our previous research also proved that tinnitus can result in significant alterations in brain white matter (WM) microstructure (Chen et al., 2020).

Indeed, microstructural changes in the brain have also been reported in some tinnitus studies (Tae et al., 2018; Besteher et al., 2019). Different studies have used different methods of measuring microstructure. Voxel-based morphometry (VBM) is a neuroimaging technique that investigates focal differences in brain anatomy (Nemoto, 2017); it can quantitatively detect the volume of brain tissue at the voxel level, reflecting the differences in the components and characteristics of brain tissue in local brain regions of different groups or individuals It can quantitatively detect the volume of brain tissue at the voxel level, reflecting the differences in brain tissue composition and characteristics in different groups or individual brain regions (Ashburner and Friston, 2000). Currently, VBM has been used increasingly widely to describe microstructural changes in the brain in tinnitus patients (Husain et al., 2011; Meyer et al., 2016). A meta-analysis revealed structural alterations in the brain of tinnitus patients in the superior temporal gyrus, middle temporal gyrus (MTG), angular gyrus, caudate nucleus, superior frontal gyrus, and supplementary motor area (Cheng et al., 2020). Our previous study also showed that compared with normal controls, patients with unilateral pulsatile tinnitus have a significantly increased gray matter (GM) volume in the bilateral superior temporal gyri (Liu et al., 2018). At the same time, structural brain networks (structural covariance networks, SCNs) were widely used in behavioral research and other diseases (Drenthen et al., 2018; Richmond et al., 2019). However, this method was not used in the evaluation of the treatment efficacy of tinnitus. In this study, SCNs were obtained using graph theoretical analysis. After obtaining the volume of GM in the first step, we continued to analyze its SCNs, to further evaluate the results.

A deep understanding of the functional and anatomical changes of the brain is a key factor for the effective treatment of tinnitus. Many treatment modalities have been applied to tinnitus patients, such as those treated with repetitive transcranial magnetic stimulation (rTMS; Poeppl et al., 2018), drug therapy (Zenner et al., 2017), tinnitus counseling, and cognitive-behavioral therapy (CBT; Langguth et al., 2013), hearing aids (Yakunina et al., 2019), cochlear implants (Olze, 2015), and tinnitus retraining therapy (Lee et al., 2019). Krick applied music therapy to observe patients’ GM volume. After the Heidelberg model of music therapy intervention, the GM of the precuneus, medial superior frontal areas, and auditory cortex increased in acute tinnitus patients accompanied by significantly decreased tinnitus-related distress (Krick et al., 2015). In recent years, narrowband-noise sound therapy is currently one of the common methods for tinnitus (Henry et al., 2002). Our previous studies have demonstrated functional changes in the brain with this sound therapy (Han et al., 2019a,b). However, we only found a few related reports on the morphological changes before and after sound therapy (Krick et al., 2015, 2017).

In this study, tinnitus patients who underwent 24 weeks of narrowband-noise sound therapy were enrolled. VBM and SCNs were applied to analyze the anatomical changes in the brain in patients before sound therapy and after sound therapy as well as to acquire data from healthy controls (HCs) at baseline and at 24 weeks to explore the morphological features and network alterations. We hypothesize that the brain regions associated with tinnitus, involving auditory, attentional, subcortical systems, and other regions, especially the superior temporal gyrus, may show volume and network alterations after sound therapy. This study will help provide deeper insight into the changes in the brain after long-term treatment for tinnitus from a neuroanatomical perspective.

## Materials and Methods

### Participants

All patients and healthy volunteers were recruited in our institution. In this study, 33 patients with idiopathic tinnitus were enrolled. The tinnitus sound was described as a persistent, high-pitched sound in both ears. The inclusion criteria and the exclusion criteria included were the same as our previous study (Han et al., 2019a,b). The characteristics of the subjects are presented in Table 1.

**Table 1:** Demographic and clinical characteristics of participants.

Characteristics	Tinnitus patients	Tinnitus patients	Healthy controls	Healthy controls	*P*-value
	(baseline, *n* = 33)	(24th week, *n* = 33)	(baseline, *n* = 26)	(24th week, *n* = 26)	
Age (years, $\bar{x}$ ± s)	48.2 ± 12.4		47.3 ± 9.6		0.745^a^
Gender (male/female)	23/10		15/11		>0.99^b^
Handedness	33 right-handed		26 right-handed		>0.99^a^
Tinnitus duration (months)	≥6 and ≤48		NA		NA
Tinnitus pitch	250–8,000 Hz				NA
THI score	52.5 ± 44.3	37.3 ± 20.9	NA	NA	0.011^c^
△THI score	15.3 ± 32.8	NA	NA	NA	NA
Normal hearing	All		All		NA

*Data are presented as mean ± standard deviation for all variables except gender. THI, Tinnitus Handicap Inventory. ΔTHI, THIpre − THIpost. NA, not applicable. ^a^Two-sample *t*-tests. ^b^Chi-square test. ^c^Paired-samples *t*-tests*.

This research involved human participants. All authors have declared that this research was approved by the Institutional Review Board (IRB). This study was approved by the ethics committees of our research institution (Beijing Friendship Hospital, Capital Medical University, 2016-P2-012). Written informed consent was obtained from all study subjects.

### Sound Therapy and Clinical Evaluation

First, to characterize the tinnitus and prepare for treatment, the audiologists in our group examined all the patients for tinnitus loudness matching, pitch matching, minimum masking level, and residual inhibition. Then, we applied narrowband-noise (that was used for treatment) to treat tinnitus for 24 weeks, 20 min each time, three times per day. For each tinnitus patient, the loudness of sound for treatment was set as L + 5 dB. The frequency was set as a 1 kHz narrowband when setting Tf as the middle point of the delivered sound (Tf ± 0.5 kHz; for example, Tf = 4 kHz, low sound cut = 3.5 kHz, high sound cut = 4.5 kHz).

We used the Tinnitus Handicap Inventory (THI) scores to assess the severity of tinnitus before and after treatment. In our study, consistent with prior research, a reduction in THI scores to 16 points or a reduction of 17 points or more was considered an effective treatment (Zeman et al., 2011). The HC group was not given any kind of sound during the study.

### Data Acquisition and Data Preprocessing

For each patient, to evaluate the change in brain activity under treatment, structural MRI data were collected at baseline and the end of therapy (24th week). The HC group was also scanned at baseline as well as at the 24th week. Images were obtained using a 3.0T MRI system (Prisma, Siemens, Erlangen, Germany) with a 64-channel phase-array head coil. During the scanning process, we used tight but comfortable foam padding to minimize head motion and earplugs to reduce scanner noise. Using a 3D magnetization-prepared rapid gradient-echo sequence (MP-RAGE), we obtained high-resolution three-dimensional (3D) structural T1-weighted images. The parameters were as follows: repetition time (TR) = 2,530 ms, echo time (TE) = 2.98 ms, inversion time (TI) = 1,100 ms, FA = 7°, number of slices = 192, slice thickness = 1 mm, bandwidth = 240 Hz/Px, field of view (FOV) = 256 mm × 256 mm, and matrix = 256 × 256, resulting in an isotropic voxel size of 1 mm × 1 mm × 1 mm.

Image preprocessing was performed with the VBM8 toolbox in the Statistical Parametric Mapping (SPM) software package (version 12)^1^. SPM 12 was installed in MATLAB 2016a (Math Works, Natick, MA, USA). The procedures for image preprocessing were the same as our previous research (Liu et al., 2018). Briefly, the normalized GM and white matter components were modulated to generate the relative gray matter volume (GMV) and white matter volume (WMV) by multiplying by the nonlinear part of the deformation field at the Diffeomorphic Anatomical Registration through Exponentiated Lie algebra (DARTEL) step. Only the GM images were analyzed in this study. The modulated GM images were smoothed with a 6 mm full width at half maximum (FWHM) isotropic Gaussian kernel. Finally, the smoothed GM images were resampled to a 3 mm × 3 mm × 3 mm voxel size for statistical analysis.

### Construction of SCNs

In this study, GM volume served as the morphological measure, and Pearson correlation was used to compute structural covariance. First, VBM8 software was used for structural image segmentation. We put GM volume maps of patients before and after treatment in one folder and maps from HCs in another folder. The GM volume value was extracted using DPABI software^2^. Second, the automated anatomical labeling (AAL) atlas was used to divide the whole brain into 90 cortical and subcortical regions of interest (Tzourio-Mazoyer et al., 2002), and each was considered a network node. Edges were defined as the Pearson Correlation coefficients of GMV of different brain regions. Last, we used the Brain Connectivity Toolbox software (Rubinov and Sporns, 2010) to construct SCNs in MATLAB version R2017a.

### SCN Analysis

We used binarized graphs to calculate global properties and local properties and calculate the area under the curve (AUC) for each property over the sparsity range. The global property was defined as the average inverse of the characteristic path length (Bullmore and Sporns, 2009). The global properties included the clustering coefficient, global efficiency, small-world properties, and shortest path length. Local (nodal) structural alterations were evaluated based on the local efficiency, degree centrality, and betweenness centrality of each region (Fortanier et al., 2019). Nodal efficiency measured the global efficiency of parallel information transfer in a network. Degree centrality is the number of nodes directly connected to the node, which measures the importance of a single node in the network. Betweenness centrality examines the contribution of each node to the shortest path between all other pairs of points (Ravasz and Barabasi, 2003). The degree centrality and betweenness centrality of nodes reflect the importance of nodes in information transfer.

### Statistical Analysis

Demographic data were compared through two-sample *t*-tests and paired two-sample *t*-tests using SPSS 19.0 software (SPSS, Inc., Chicago, IL, USA). *P* values < 0.05 were considered statistically significant. Longitudinal changes in the THI score were also analyzed by using paired two-sample *t-tests*.

For VBM data, to determine the group × time interaction effect between the two groups and the two scans, the main effects of group (the tinnitus patient group and the HC group) and time (baseline and 24-week follow-up period), two-way mixed model analysis of variance (ANOVA) and *post hoc* analyses were performed. An *F*-value of VBM analysis and a *P*-value of SCN analysis less than 0.05 were considered statistically significant [false discovery rate (FDR) corrected].

In *post hoc* analyses, Student-Newman-Keuls (SNK) tests were used for pairwise comparisons.

To prove our hypothesis, Pearson’s correlation analyses were further conducted to investigate the relationship between the change in GM volume and 24 weeks of sound therapy (△THI score = THIpre − THIpost). *P* < 0.05 was set as the threshold to determine significance. The GM volume results were visualized with BrainNet Viewer^3^ (Xia et al., 2013). Pearson’s correlation analysis was performed using SPSS 19 software (SPSS, Inc., Chicago, IL, USA) between the THI scores.

For SCNs, we used two-sample *t*-tests to construct a network of patients with tinnitus (baseline and after 24-week treatment) and a healthy group (baseline and after 24-week scan). Then, the graph theory index of the covariant brain network was calculated, and a permutation test (1,000 times) was performed on the graph theory index of the two groups. We applied an FDR of 5% to adjust for the multiple comparisons of mean local and global efficiency across the between-group contrasts before and after 24 weeks of treatment.

## Results

### Demographics and Behaviors of Study Participants

In this study, we enrolled 33 patients with idiopathic tinnitus, and we applied VBM to analyze the GM volume and network changes in the brain in this group before and after sound therapy. Concurrently, 26 HC individuals were enrolled. Each group of subjects was age-, sex-, and handedness-matched (Table 1). THI scores were acquired before and after sound therapy. In the data preprocessing step, none of the subjects were excluded according to the head motion criteria. Significant longitudinal decreases in THI scores were observed. The results are summarized in Table 1.

### Statistical Analysis Results

#### Brain Structural Changes Between the Patient Group and HC Group at Baseline and Either After Treatment or 24 Weeks, Respectively, and Between the Patients Before and After Treatment

**Figure 1:**
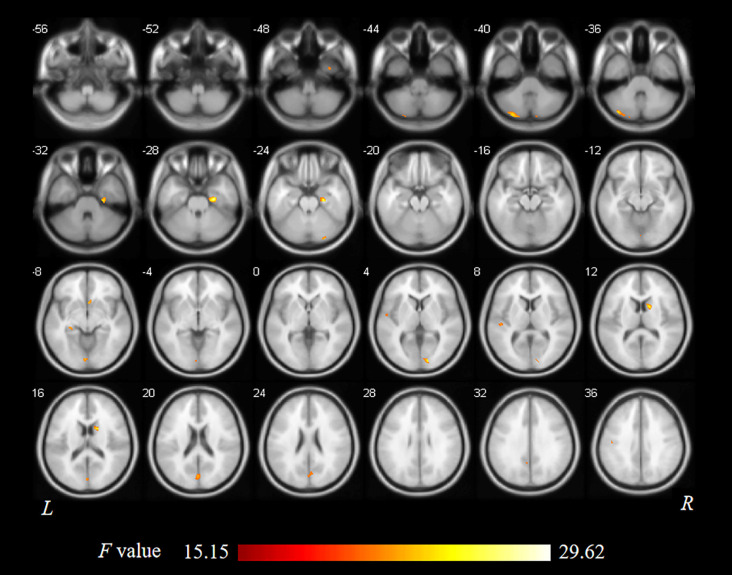
Analysis of variance (ANOVA) differences in gray matter volume (GMV) changed among patients at baseline, patients after 24 weeks of sound treatment, healthy controls (HCs) at baseline, and HCs after 24 weeks (*P* < 0.05; L, left; R, right). The results showed differences in gray matter (GM) volume in the right parahippocampus gyrus, right caudate, left superior temporal gyrus, left cuneus gyrus, and right calcarine gyrus, which is shown in red. L, left; R, right.

As shown in Figure 1 and Table 2, statistical analysis results demonstrated significant differences in GM volume among the tinnitus patients before sound therapy (baseline), tinnitus patients after sound therapy (24 weeks), HC individuals at baseline, and HC individuals after 24 weeks. These brain regions included the right parahippocampus gyrus, right caudate, left superior temporal gyrus, left cuneus gyrus, and right calcarine gyrus (without multiple corrections).

**Table 2:** Regions showed a significant difference in volumes between idiopathic tinnitus patients before and after 24 weeks of sound therapy and the controls.

Brain regions		Peak location			Peak *F*-score	Voxel numbers
		x	y	z		
Parahippocampus gyrus	R	25	−18	−25	29.62	541
Caudate	R	16	7	14	20.04	438
Superior temporal gyrus	L	−47	−23	8	15.15	117
Cuneus gyrus	L	0	−77	22	15.96	371
Calcarine gyrus	R	14	−88	6	20.57	441

**Figure 2:**
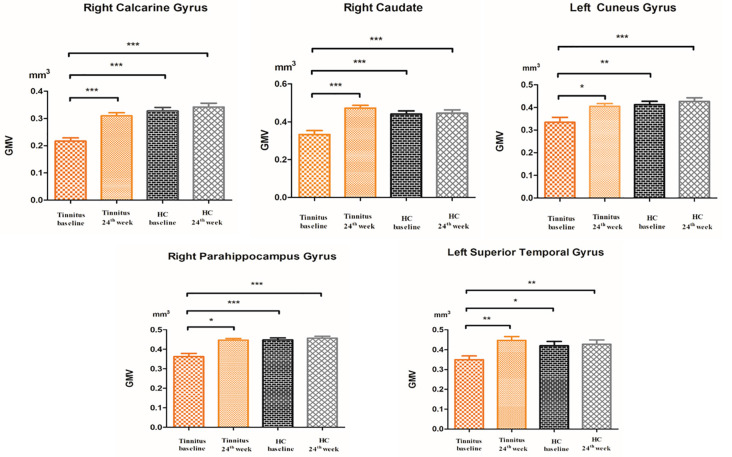
The *post hoc* analysis showed that the GMV changed among the baseline, 24-week sound treatment, HC baseline, and HC 24-week groups. Compared with the tinnitus baseline group, the 24-week sound therapy tinnitus group demonstrated a significantly higher GM volume in all of the regions as this figure (*P < 0.05, **P < 0.01, ***P < 0.001), compared with the tinnitus baseline group, the HC health baseline group and the 24-week HC group demonstrated a significantly higher GM volume in all of the regions as this figure (*P < 0.05, **P < 0.01, ***P < 0.001). The *P*-values are not corrected for multiple comparisons.

Compared to participants in the HC baseline group and HC 24-week group, significantly decreased GM volume was found in the right parahippocampus gyrus, right caudate, left superior temporal gyrus, left cuneus gyrus, and right calcarine gyrus of the participants in the tinnitus baseline group (Table 2 and Figure 2).

Compared with the tinnitus baseline group, the 24-week sound therapy tinnitus group demonstrated a significantly higher GM volume in all of the regions mentioned above. Compared with the GM in the HC baseline group, the GM in the HC 24-week group did not reach statistical significance in these brain regions (Table 2 and Figure 2).

Compared with the HC baseline group and the HC 24-week group, the tinnitus sound therapy group demonstrated slightly lower GM volume in the right calcarine gyrus and left cuneus gyrus and slightly higher volume in the right caudate and left superior temporal gyrus; however, these differences did not reach statistical significance (Table 2 and Figure 2).

#### SCN Changes Between the Patient and HC Groups at Baseline and Either After Treatment or After 24 Weeks, Respectively, and Between Patients Before and After Treatment

**Figure 3:**
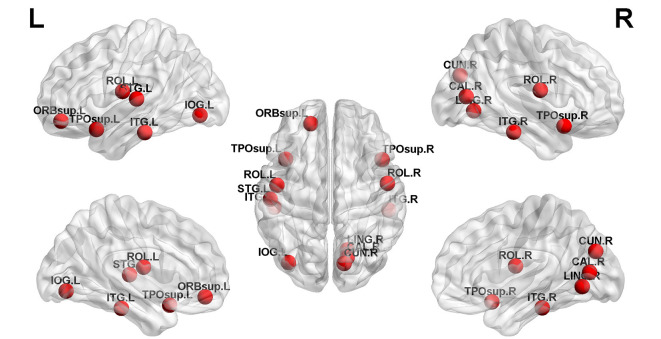
Twelve brain regions reached statistical significance in nodal efficiency and nodal betweenness centrality of the automated anatomical labeling (AAL)-90 structural covariance network (SCN) in patients before and after treatment, in HCs at baseline, and in HCs after 24 weeks (red ball, *P* < 0.05). The results were produced using permutation testing and visualized using the BrainNet Viewer (NKLCNL, Beijing Normal University). Three-dimensional representations (left: lateral and medial view of the left hemisphere; center: dorsal view; right: lateral and medial view of the right hemisphere) show between-group differences in nodal efficiency, degree centrality, and nodal betweenness centrality, according to their centroid stereotaxic coordinates. A list of anatomical labels for the nodes is in Table 3.

**Figure 4:**
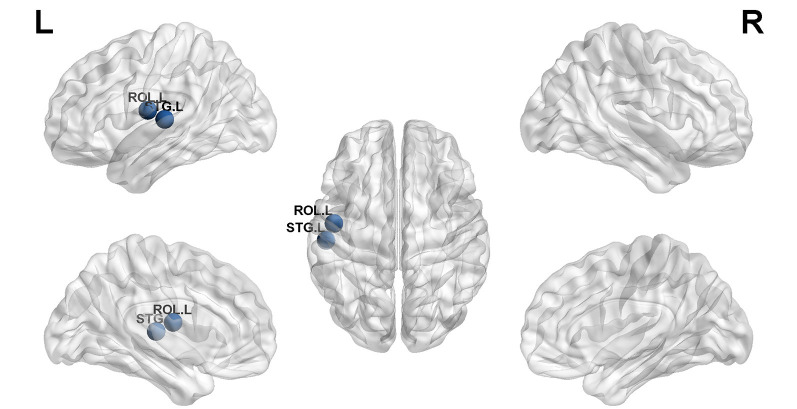
The left superior temporal gyrus and left rolandic operculum were significantly different in nodal efficiency, nodal degree centrality, and nodal betweenness centrality after FDR correction (blue ball, *P* < 0.05).

Structural covariance, a measure of structural brain connectivity, was measured between all pairs of cortical regions. We calculated the AUC for each network metric, and the AUC provided a summarized scalar for the topological characterization of brain networks. The results showed that there was statistical significance in 12 brain regions (Table 3 and Figure 3), including nonauditory-related and auditory-related brain regions, such as the bilateral rolandic operculum and left superior temporal gyrus, and all 12 regions combined. The left superior temporal gyrus and left rolandic operculum were significantly different in nodal efficiency, nodal degree centrality, and nodal betweenness centrality after FDR correction (Table 3 and Figure 4). Combining the above results, we found that only the left superior temporal gyrus showed significant differences in GM volume and SCNs.

**Table 3:** Regions showed a significant difference in network (*P* < 0.05, FDR corrected, 1,000 permutations).

Characteristics	AAL Brain regions	*P*-value	AAL Brain regions	*P*-value
Nodal Efficiency	5_Frontal_Sup_Orb_L	0.001	17_Rolandic_Oper_L	0.000^a^
	18_Rolandic_Oper_R	0.002	44_Calcarine_R	0.024
	46_Cuneus_R	0.029	48_Lingual_R	0.049
	53_Occipital_Inf_L	0.01	81_Temporal_Sup_L	0.000^a^
	83_Temporal_Pole_Sup_L	0.023	84_Temporal_Pole_Sup_R	0.005
	89_Temporal_Inf_L	0.028	90_Temporal_Inf_R	0.043
Nodal DC	5_Frontal_Sup_Orb_L	0.001	17_Rolandic_Oper_L	0.000^a^
	18_Rolandic_Oper_R	0.002	44_Calcarine_R	0.024
	46_Cuneus_R	0.029	48_Lingual_R	0.049
	53_Occipital_Inf_L	0.023	81_Temporal_Sup_L	0.000^a^
	83_Temporal_Pole_Sup_L	0.023	84_Temporal_Pole_Sup_R	0.005
	89_Temporal_Inf_L	0.028	90_Temporal_Inf_R	0.043
Nodal BC	5_Frontal_Sup_Orb_L	0.001	17_Rolandic_Oper_L	0.000^a^
	18_Rolandic_Oper_R	0.002	44_Calcarine_R	0.024
	46_Cuneus_R	0.029	48_Lingual_R	0.049
	53_Occipital_Inf_L	0.01	81_Temporal_Sup_L	0.000^a^
	83_Temporal_Pole_Sup_L	0.023	84_Temporal_Pole_Sup_R	0.005
	89_Temporal_Inf_L	0.028	90_Temporal_Inf_R	0.043

*^a^FDR corrected; DC, degree Centrality; BC, betweenness centrality*.

#### Correlation

The decreased THI score and GM volume change between these five brain regions were not correlated.

## Discussion

This is a meaningful longitudinal investigation. We observe the changes in GM volume and SCNs in tinnitus patients at baseline and after 24 weeks of sound therapy. In this study, we found that tinnitus patients had structural changes in the brain after treatment. Anatomical changes in the brain were found in patients before and after sound therapy, mainly in the right parahippocampus gyrus, right caudate left superior temporal gyrus left cuneus gyrus, and right calcarine gyrus. The results of the SCNs provided crucial information for understanding the network interactions between the whole brain and therapy in tinnitus. To a certain extent, these brain regions can be used as neurobiological targets for tinnitus treatment.

### Nonauditory-Related Structural Brain Alterations and the Network Performance Between Patients and HCs and Between Patients Before and After Sound Therapy

We observed a significant increase in GM volume in the right parahippocampus gyrus, right caudate, left cuneus gyrus, and right calcarine gyrus in the patients after treatment compared with baseline. The parahippocampal gyrus is regarded to be critical to emotional processing and auditory information storage in tinnitus patients; therefore, some studies have noticed that the parahippocampal gyrus is related to perception in tinnitus patients (Leaver et al., 2016; Vanneste and De Ridder, 2016). A previous GM study found two major group differences that decreased cortical thickness in the left parahippocampal gyrus in patients with severe tinnitus (Schmidt et al., 2018). Our results showed that compared with the tinnitus baseline group, the 24-week sound therapy group demonstrated a significantly higher GM volume in the parahippocampal gyrus. This result showed that after a long period of treatment, the hearing information storage and emotion of tinnitus patients had a certain degree of recovery. The calcarine gyrus is an important part of the primary visual cortex and the main relay station for transmitting retinal signals; thus, changes in the calcarine gyrus may result from patients attending to phantom auditory sensations and having the visual areas contemporaneously activated (Zhou et al., 2019). Our previous research has also shown that the local activity and functional connectivity of the primary auditory cortex were enhanced (Lv et al., 2020).

In the SCN results, we found that there was a significant difference in the rolandic operculum of nodal efficiency, degree centrality, and betweenness centrality. A study speculated that overactivity in the rolandic operculum was associated with middle ear proprioception, and changes in this brain region may suggest that tinnitus could arise as a proprioceptive illusion associated with widespread emotional and somatosensory dysfunction (Job et al., 2012). Our results further confirm this inference. Although these SCN changes may not be as significant as functional changes, the results also reflect the efficacy of sound therapy in local network properties to a certain extent.

### Auditory-Related Brain Structural Alterations and the Network Performance Between Patients and HCs and Between Patients Before and After Sound Therapy

The auditory cortex can be divided into the primary auditory cortex, secondary auditory cortex, and auditory association cortex. Our results indicated increased GM volume in the left superior temporal gyrus, which is the auditory network, mainly overlapping with the auditory association cortex. The abnormal parts of the auditory cortex of tinnitus patients, mainly the auditory association cortex, are relatively sensitive compared to other parts (Chen et al., 2014; Zhang et al., 2015; Lv et al., 2016a). The left superior temporal gyrus in degree centrality reached a significant difference, indicating the importance of a single node (left superior temporal gyrus) in the network.

Regarding the morphological and functional changes associated with tinnitus, most previous studies and our recent research have focused on functional aspects (Minami et al., 2018). In this study, the 24-week treatment group showed high levels of auditory-related GM volume, but the tinnitus baseline group showed less auditory-related GM volume, which suggests that the associations between networks defined as being within the auditory-related network architecture were generally stronger in the 24-week treatment group. Previous studies have mentioned functional issues but performed fewer structural analyses (Han et al., 2019b; Berlot et al., 2020). There are structural changes in tinnitus patients, and these structural changes will affect structure after treatment (Cheng et al., 2020); therefore, it is necessary to pay more attention to the structural changes associated with tinnitus patients. The results of this study are important supplements for original research. Therefore, whether in VBM or SCN analyses, the superior temporal gyrus can be used as one of the important structural brain areas to measure the effect of tinnitus treatment.

Compared with previous research on tinnitus, in this study, the aforementioned brain regions could represent new neuroanatomical features of patients with tinnitus. In particular, the superior temporal gyrus, whether in VBM or SCN analyses, can be used as an important structural brain area to measure the effect of tinnitus treatment. Accordingly, combined VBM and SCN analyses can provide novel tools to examine complex network properties of the intact and diseased brain. The two modalities are complementary.

A suitable treatment method and the correct treatment time are the keys to achieving curative effects. In our study, we applied narrow band noise sound therapy with a relatively long treatment time and a relatively better treatment effect. A previous study with the Heidelberg model of music therapy analyzed the average treatment time to be approximately 2 weeks (Krick et al., 2015) and found GM volume changes in the precuneus, medial superior frontal areas, and auditory cortex. The analysis in our study was performed for 6 months of treatment with narrowband-noise sound therapy. The morphological changes that may be found are different from those of previous studies, which is supported by our results. However, for different results, we should consider different opinions, which will help to obtain a balanced view of our research.

### Limitations

There are several limitations to this study. First, the current study is an exploratory study with microstructure changes in GM, and it is difficult to obtain a significant result with small sample size. Therefore, a threshold of *p* < 0.001 (uncorrected for multiple comparisons) was applied in the condition group comparisons based on the stringency of the group contrasts used in this study. In contrast to functional changes, it is difficult for the microstructure itself to change significantly within a relatively short period. Therefore, we believe that this result can explain the corresponding scientific problem to a certain extent. Further studies should also include a larger sample size to avoid these problems. Second, there have been some studies that have applied VBM to further explore the characteristics of tinnitus, but the reported results are not completely consistent. Maybe the methodological differences lead to heterogeneous results (Scott-Wittenborn et al., 2017). We should further pay attention to heterogeneity in future studies. Third, the tinnitus patients included in this study did not have significant hearing loss. Last, SCNs have been evaluated concerning other clinical diseases, but there are few studies on tinnitus, so we should perform more in-depth research in the future.

## Conclusion

This study analyzed the anatomical changes in tinnitus patients before and after treatment for 6 months. The effect of sound therapy included alterations in brain volume, especially in the left superior temporal lobe. Combined VBM and SCNs can potentially provide us with a better understanding of the neuroanatomical and pathophysiological mechanisms before and after sound therapy in tinnitus patients. Sound therapy had a normalizing effect on tinnitus patients.

## Data Availability Statement

The original contributions presented in the study are included in the article, further inquiries can be directed to the corresponding author/s.

## Ethics Statement

The studies involving human participants were reviewed and approved by the ethics committees of our research institution (Beijing Friendship Hospital, Capital Medical University, 2016-P2-012). The patients/participants provided their written informed consent to participate in this study. Written informed consent was obtained from the individual(s) for the publication of any potentially identifiable images or data included in this article.

## Author Contributions

XW designed the experiments, performed the statistical analysis, and wrote the manuscript. QC conducted the statistical analysis. PfZ contributed to the manuscript revision. HL, ZW, and CL collected the data. SG, ZY, and ZcW are guarantors of this work. HL and ZcW are the corresponding authors of this manuscript. They have full access to all the data in the study and take responsibility for the integrity of the data and the accuracy of the data analysis. All authors contributed to the article and approved the submitted version.

## Conflict of Interest

The authors declare that the research was conducted in the absence of any commercial or financial relationships that could be construed as a potential conflict of interest.

## Footnotes

^1^https://www.fil.ion.ucl.ac.uk/spm

^2^http://rfmri.org/dpabi

^3^http://www.nitrc.org/projects/bnv/
